# Spontaneous cyclical fluctuation in respiratory minute volume during prone position ventilation in a patient with COVID-19

**DOI:** 10.1186/s13054-022-04072-3

**Published:** 2022-07-01

**Authors:** Olivier van Minnen, Joep M. Droogh

**Affiliations:** Department of Critical Care, University Medical Center Groningen, University of Groningen, Groningen, The Netherlands

**Keywords:** Mechanical ventilation, Prone position, ARDS

Dear Editor,

Prone positioning of patients with acute respiratory distress syndrome (ARDS) has been used for many years. During the ongoing COVID-19 pandemic, prone position has largely been adopted by clinicians. Improvement in oxygenation and reduction in mortality are the main reasons to apply prone position in ARDS patients. The change from supine to prone position gives a better distribution of the gas/tissue ratio and a more homogeneous distribution of lung stress and damage. The reason for the reduced mortality is less overdistention of the lungs and less cyclical opening and closing of the alveoli [1].

A 74-year-old male patient, who was admitted to the intensive care unit (ICU) for respiratory failure due to severe COVID-19 ARDS, required invasive mechanical ventilation in prone position because of severely impaired oxygenation. The patient was treated with pressure-controlled ventilation, was deeply sedated and continuous neuromuscular blockade was achieved. We noticed a spontaneous cyclical fluctuation in respiratory minute volume and expiratory tidal volume with constant ventilator settings. The expiratory tidal volume fluctuated between 306 and 428 ml (mean 380.94 ml, standard deviation (SD) 27.15 ml) every 7.5 min. The respiratory minute volume fluctuated between 7.9 l/min and 10.9 l/min (mean 9.90 l/min, SD 0.70 l/min).

Patients admitted to our ICU are positioned on an air-cushion anti-decubitus mattress (Wissner-Bosserhoff, Vituoso 2®). This mattress consists of 17 air-filled cells and uses alternating pressure therapy, in which 1/3 of the cells alternately deflate and inflate every 7.5 min to prevent pressure ulcers of the skin. We hypothesized that the fluctuation in tidal volumes and respiratory minute volume were caused by the alternating inflation of the cells of the mattress.

To determine the influence of the alternating pressure therapy on the tidal volume, we set the mattress to the 'max inflate' mode, which is normally used during patient care. All cells are fully inflated in this modality. During the use of the max inflate modality, we observed a stable respiratory minute volume (min 8.9 l/min, max 10.2 l/min, Mean 9.97 l/min, SD 0.24 l/min) and expiratory tidal volume (min 372.6 ml, max 398.6 ml, Mean 385.62, SD 6.22), which started to fluctuate again after restarting the alternating pressure therapy (Fig. 1). These findings were not observed in supine position.

**Fig. 1:**
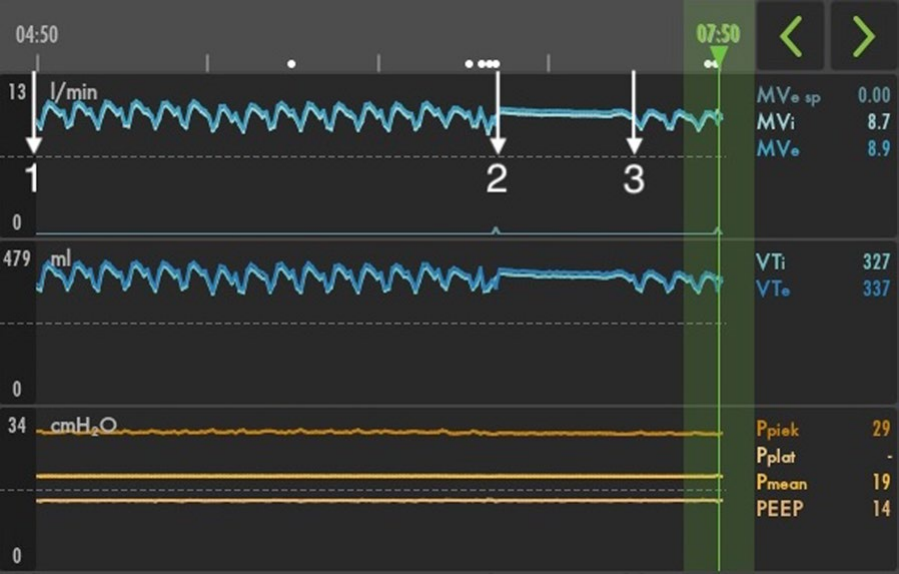
Respiratory minute volume and expiratory tidal volume trend log of a 3-h period. 1: Fluctuation in respiratory minute and expiratory tidal volume in 7.5-min intervals during alternating pressure therapy of the mattress; 2: Stable respiratory minute and expiratory tidal volume during the max-inflate modality of the mattress; 3: Reproduced fluctuation in respiratory minute and expiratory tidal volume after reintroducing alternating pressure therapy

A possible explanation for the fluctuation in respiratory minute volume might be the pressure changes on the thorax and abdomen caused by the mattress, resulting in a reduction of chest wall compliance. This emphasizes the need for optimal positioning of patients in the prone position. Since driving pressures in severely ARDS patients are preferably set to values below 15 cm H2O and as a consequence, pH values are accepted down to 7.22 accordingly [2], minute ventilation volumes should be closely monitored. Therefore, one should be aware of the described changes in minute ventilation volumes due to the mattress settings. On the other hand, some preclinical models showed improved oxygenation and reduced histological lung damage when variable tidal volumes were used during pressure control ventilation in ARDS [3–5]. Therefore, the fluctuation in tidal volumes caused by the mattress could have had beneficial effects for this patient. However, more research is needed.

## Data Availability

The dataset used and analyzed during this current study are available from the corresponding author on reasonable request.
